# Assessing the information content of structural and protein–ligand interaction representations for the classification of kinase inhibitor binding modes via machine learning and active learning

**DOI:** 10.1186/s13321-020-00434-7

**Published:** 2020-05-24

**Authors:** Raquel Rodríguez-Pérez, Filip Miljković, Jürgen Bajorath

**Affiliations:** Department of Life Science Informatics, B-IT, LIMES Program Unit Chemical Biology and Medicinal Chemistry, Rheinische Friedrich-Wilhelms-Universität, Endenicher Allee 19c, 53115 Bonn, Germany

**Keywords:** Active learning, Machine learning, Atom environment fingerprints, Interaction fingerprints, Kinase inhibitors, X-ray structures, Binding modes

## Abstract

For kinase inhibitors, X-ray crystallography has revealed different types of binding modes. Currently, more than 2000 kinase inhibitors with known binding modes are available, which makes it possible to derive and test machine learning models for the prediction of inhibitors with different binding modes. We have addressed this prediction task to evaluate and compare the information content of distinct molecular representations including protein–ligand interaction fingerprints (IFPs) and compound structure-based structural fingerprints (i.e., atom environment/fragment fingerprints). IFPs were designed to capture binding mode-specific interaction patterns at different resolution levels. Accurate predictions of kinase inhibitor binding modes were achieved with random forests using both representations. The performance of IFPs was consistently superior to atom environment fingerprints, albeit only by less than 10%. An active learning strategy applying information entropy-based selection of training instances was applied as a diagnostic approach to assess the relative information content of distinct representations. IFPs were found to capture more binding mode-relevant information than atom environment fingerprints, leading to highly predictive models even when training instances were randomly selected. By contrast, for atom environment fingerprints, the derivation of accurate models via active learning depended on entropy-based selection of informative training compounds. Notably, higher information content of IFPs confirmed by active learning only resulted in small improvements in global prediction accuracy compared to models derived using atom environment fingerprints. For practical applications, prediction of binding modes of new kinase inhibitors on the basis of chemical structure is highly attractive.
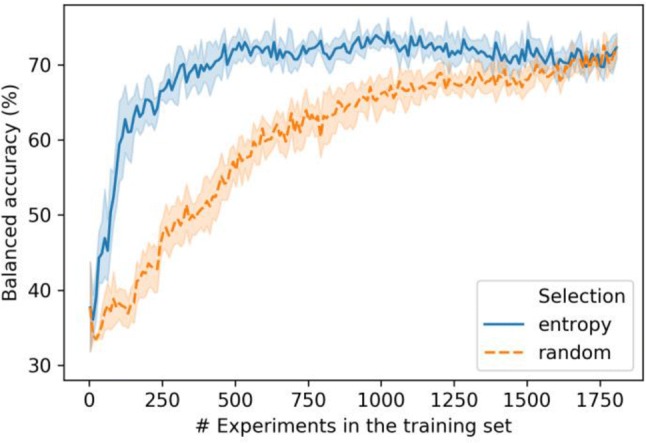

## Introduction

Volumes of publicly available kinase inhibitor data have dramatically increased in recent years, enabling systematic computer-aided investigations of activity profiles, structure–activity relationships (SARs), and promiscuity versus selectivity trends [1–4]. In addition to activity data analysis, computational approaches have also been used for predictive modeling of kinase inhibitor activity, for example, to distinguish between kinase inhibitors having high and low potency [5]. Large amounts of inhibitor data are complemented by increasing numbers of three-dimensional (3D) structures of kinase-inhibitor complexes that become available [6, 7] and enable a thorough exploration of compound binding modes and structure-assisted SAR exploration. In addition, these complexes provide templates for structure-based ligand design [8].

Distinct inhibitor binding modes revealed by X-ray crystallography depend on structural differences between the active and inactive form of kinases [9, 10]. 3D structures of kinases and inhibitor complexes revealed different activation states involving the activation loop containing the characteristic DFG tripeptide motif as well as the αC-helix in the active site region. In the active form, the activation loop is closed adopting the so-called “DFG in” conformation and the αC-helix forms a K-E salt bridge between the β3 strand and the αC-helix (“αC-helix in” conformation) [10]. The so-called type I binding mode is observed for the majority of kinase inhibitors. These compounds represent ATP site directed inhibitors and bind to the active (“DFG in/αC-helix in”) form of kinases. In addition, type II inhibitors bind to the inactive form, which is characterized by the “DFG out” and “αC-helix out” conformations. These inhibitors occupy a hydrophobic pocket adjacent to the ATP site that opens when the DFG motif adopts the “out” conformation. Another type of inhibitors targets a conformational state falling in between the active and inactive forms. These designated type I½ inhibitors bind to kinases with closed activation segment and the αC-helix out conformation (“DFG in”/“αC-helix out”). Furthermore, there are allosteric type III or IV inhibitors that bind to other regions in kinases outside their active site. Finally, bivalent and covalent inhibitors represent type V and VI, respectively [9].

Computationally, protein–ligand interactions can be accounted for by interaction fingerprints (IFPs) that are one-dimensional (1D) binary representations, in analogy to fragment fingerprints, designed to capture intermolecular interactions in complex structures [11, 12]. Accordingly, IFPs represent a “structural interaction profile” of a protein–ligand complex that can be used for organizing and visualizing interaction information as well as for similarity searching [11–13]. Application of IFPs is not limited to experimental structures as they can also be used to capture interactions in predicted ligand-target complexes, for example, complexes from docking. IFPs can then be used to rank docking poses of test compounds based on interaction similarity to reference structures [14, 15]. In some instances, compound ranking performance of residue- and atom-based IFPs was found to be superior to conventional force field-based scoring functions [13, 16]. However, IFPs might fail to detect key interactions or equally weight protein–ligand contacts that are critical or largely irrelevant for binding, which introduces noise in IFP comparisons. Moreover, IFP generation also depends on specific features of binding sites, which may restrict their general use across targets with different binding site architectures. These issues have limited widespread use of IFPs in drug design. Taking such potential limitations into consideration, a previous study attempted to predict IFPs for three target proteins on the basis of compound structures [17]. To these ends, IFPs were first calculated for complex structures. Then, neural networks were trained to predict IFPs on the basis of ligand descriptors. While these calculations supported proof-of-concept their accuracy remained limited. For the training set, an average Tanimoto coefficient (Tc) of 0.7 for original and predicted IFPs was obtained, with a rather widespread distribution. For ~ 70% of the test compounds, corresponding Tc values were at least 0.6 [17]. In general, IFPs provide a valuable format for effectively encoding protein–ligand interaction information that can be used for similarity searching or machine learning.

Recently, prediction of kinase inhibitors adopting different binding modes using machine learning on the basis of chemical structure yielded surprisingly accurate results [18]. Predictive modeling was performed for more than 2000 crystallographically characterized inhibitors that were represented using atom environment/structural fingerprints [18]. The results indicated that kinase inhibitors exhibited structural patterns that correlated with different binding modes such that accurate predictions were possible without taking target structure or ligand-target interaction information into account. While one would expect that inhibitors contain specific structural features that lead to distinct binding modes, only few such features distinguishing different types of kinase inhibitors have been elucidated so far [19]. Thus, the ability of machine learning to systematically distinguish between different types of inhibitors is thought to result from detecting structural characteristics that are difficult to recognize on the basis of expert knowledge.

Distinguishing between inhibitors with different binding modes also represents a prime application for IFPs. By design, IFPs should capture binding mode-specific interaction patterns. Since binding modes of kinase inhibitors can also be accurately predicted from chemical structure, without taking interactions into account [18], this prediction task represents an excellent test case for comparing the relevance of compound structure and target-ligand interaction information via machine learning. Moreover, with more than 2000 currently available kinase inhibitors with structurally confirmed binding modes, a much larger knowledge base can be utilized for this comparison than has been the case for many previous IFP applications using X-ray data.

In this work, the information content of compound structure and protein–ligand interaction representations has been evaluated through machine learning approaches. In addition, active learning strategies were applied as a diagnostic approach to further compare these representations and determine the number of training instances required for successful classification of kinase inhibitors with different binding modes.

## Results and discussion

### Kinase inhibitors with different binding modes

Type I, I½, and II kinase inhibitors were extracted from X-ray structures of kinase-inhibitor complexes contained in the KLIFS database [6, 7], a specialized repository for kinase structures and associated activity data, as detailed in “Methods” section. The composition of the kinase inhibitor data set is reported in Table 1.

**Table 1:** Kinase inhibitors with different binding modes

Type	# Inhibitors (%)
I	1424 (70.9%)
I½	394 (19.6%)
II	190 (9.5%)
Total	2008

The composition of the compound data set assembled from X-ray structures of kinase-inhibitor complexes is summarized

### Study design

We have aimed to compare distinct molecular and interaction representations for machine learning using different modeling strategies. For this purpose, kinase inhibitors with different binding modes were classified. This investigation was inspired by previous findings that such inhibitors could be predicted with high accuracy on the basis of chemical structure using standard machine learning approaches such as random forest (RF) [18]. These observations and the availability of large numbers of kinase inhibitors with experimentally determined binding modes provided a sound basis for a comparative study including active learning strategies to assess the information content of structural and interaction representations on a relative scale.

**Fig. 1:**
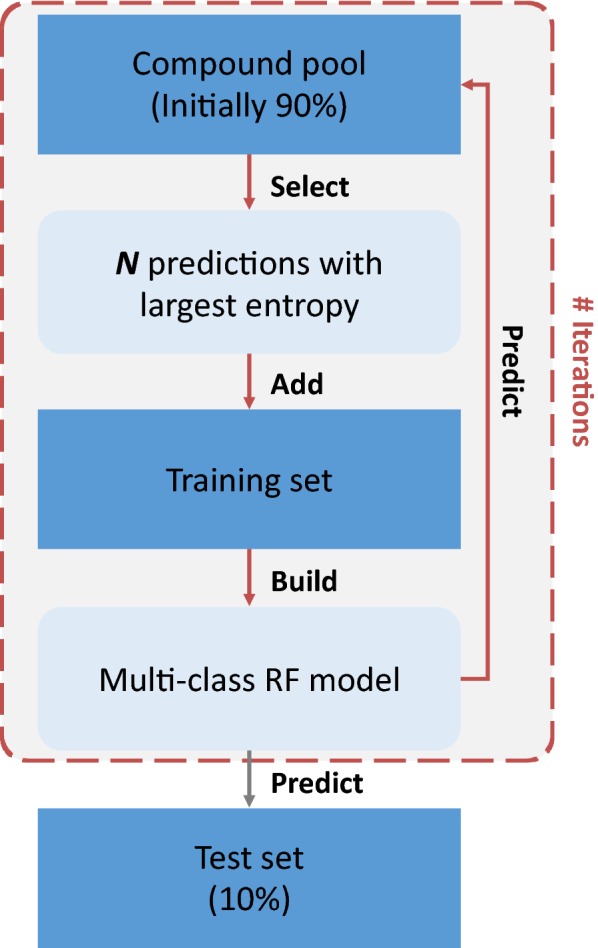
Active learning strategy. Training instances are selected randomly (first iteration) or based on an entropy criterion (subsequent iterations) after predicting pool compounds. For performance evaluation, the multi-class RF model is then used to predict the external test set

First, conventional RF models were derived using 90% of available inhibitors and applied to classify the test set containing the remaining 10% of the inhibitors. Moreover, an active learning strategy was implemented, which iteratively selects informative training instances in order to reduce training data to a required minimum. Hence, if successful, active learning reveals information that is essential for predictive modeling. Active learning employed a multi-class RF model starting with a corresponding data split for iterative sample selection and class label prediction, as illustrated in Fig. 1. Training instances were selected on the basis of information entropy from the compound pool, which initially corresponded to a 90% of the data set. The model trained with selected instances was then used to predict the test set (10%). Further details and calculation protocols are provided in the Methods section.

### Random forest predictions

Binding mode predictions were attempted with fundamentally different representations including IFPs and molecular graph-based fingerprints (see “Methods” section for details). IFPs included an 85-bit version accounting for the presence or absence of ligand interactions with 85 residue positions forming the binding site region in kinases (IFP_85), and a further expanded 595-bit version distinguishing between seven different types of interactions for inhibitors and each residue position (85 × 7; IFP_595). The 85 residues represent the complete active site region in kinases defined on the basis of many X-ray structures [6, 7]. Others have previously used smaller subsets of these residues focusing on the ATP site, which were predicted to be important for conferring kinase selectivity [20, 21]. However, in our analysis, the comprehensive representation of the binding site region was used because different inhibitor binding modes were predicted. As a representation of chemical structures, the folded (1024-bit) and unfolded (variably sized feature set) version of the extended connectivity fingerprint with bond diameter 4 (ECFP4) were generated for each inhibitor (termed ECFP4_folded and ECFP4_unfolded, respectively). ECFP4 is a topological fingerprint encoding layered atom environments.

**Fig. 2:**
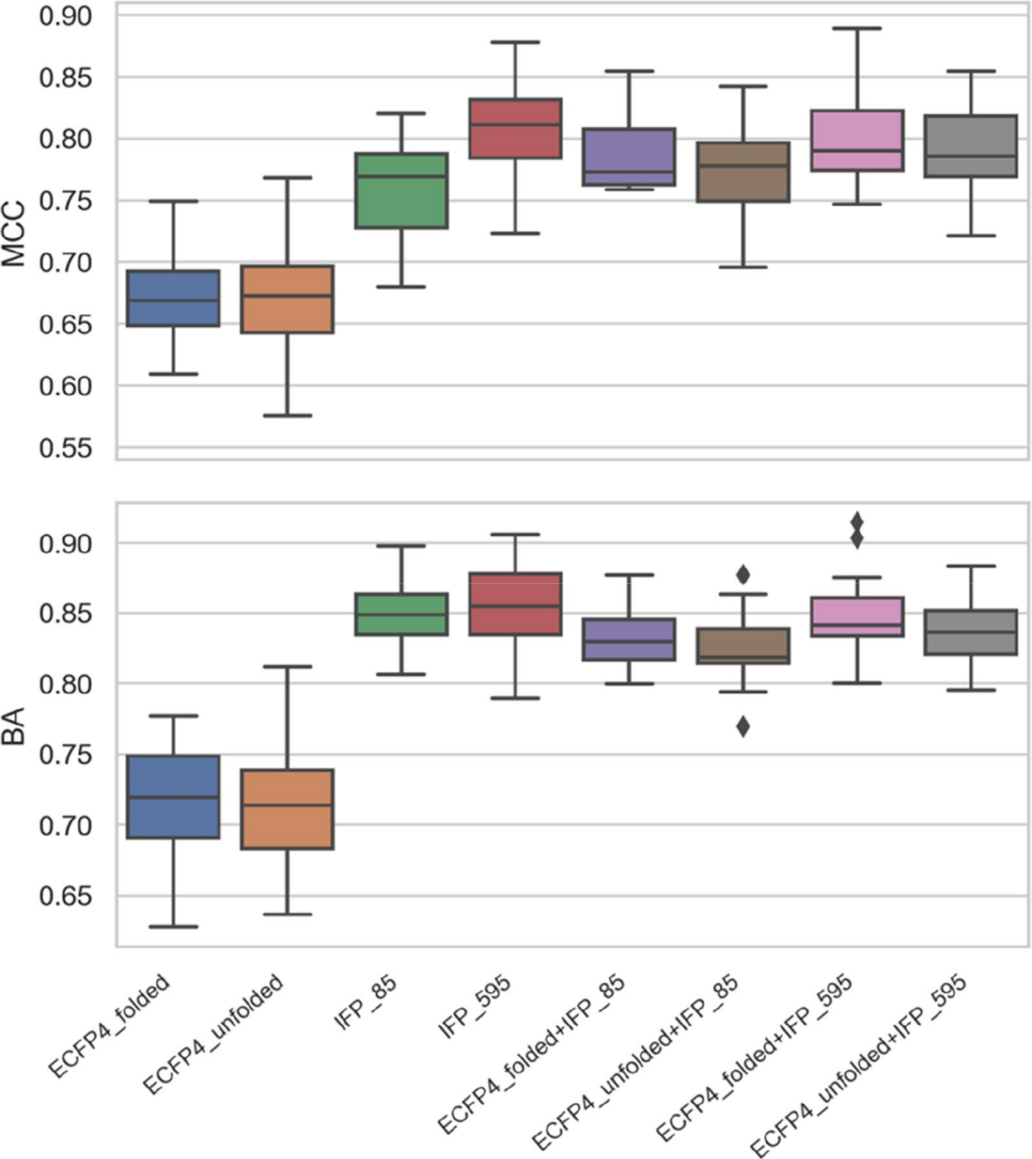
Predictive performance of random forest models on test sets. MCC and BA value distributions are reported for RF models using different representations

For classification, multi-class RF models were derived to distinguish between type I, I½, and II inhibitors. Figure 2 reports the Matthew’s correlation coefficient (MCC) and balanced accuracy (BA) values for RF models trained with both IFPs, ECFP4, and combined representations over 20 independent trials. Overall, RF models on the basis of ECFP4 yielded accurate predictions, consistent with our previous observations. This was the case for the folded and unfolded ECFP4 version, with median BA and MCC values greater than 0.70 and 0.65, respectively. However, application of IFPs further increased global prediction accuracy. IFP_85 yielded median BA and MCC values of 0.85 and 0.76, respectively. In addition, IFP_595 with further refined interaction information produced comparable BA but further increased MCC values, with a median MCC of 0.81. Compared to IFPs, model performance essentially remained constant when IFP and ECFP4 representations were combined (i.e., when fingerprints of different design were concatenated). Only very minor changes were observed that were not significant. Hence, IFP contributions mostly determined prediction accuracy and the minor fluctuations or reductions were likely due to ECFP4 feature noise in combined representations.

**Fig. 3:**
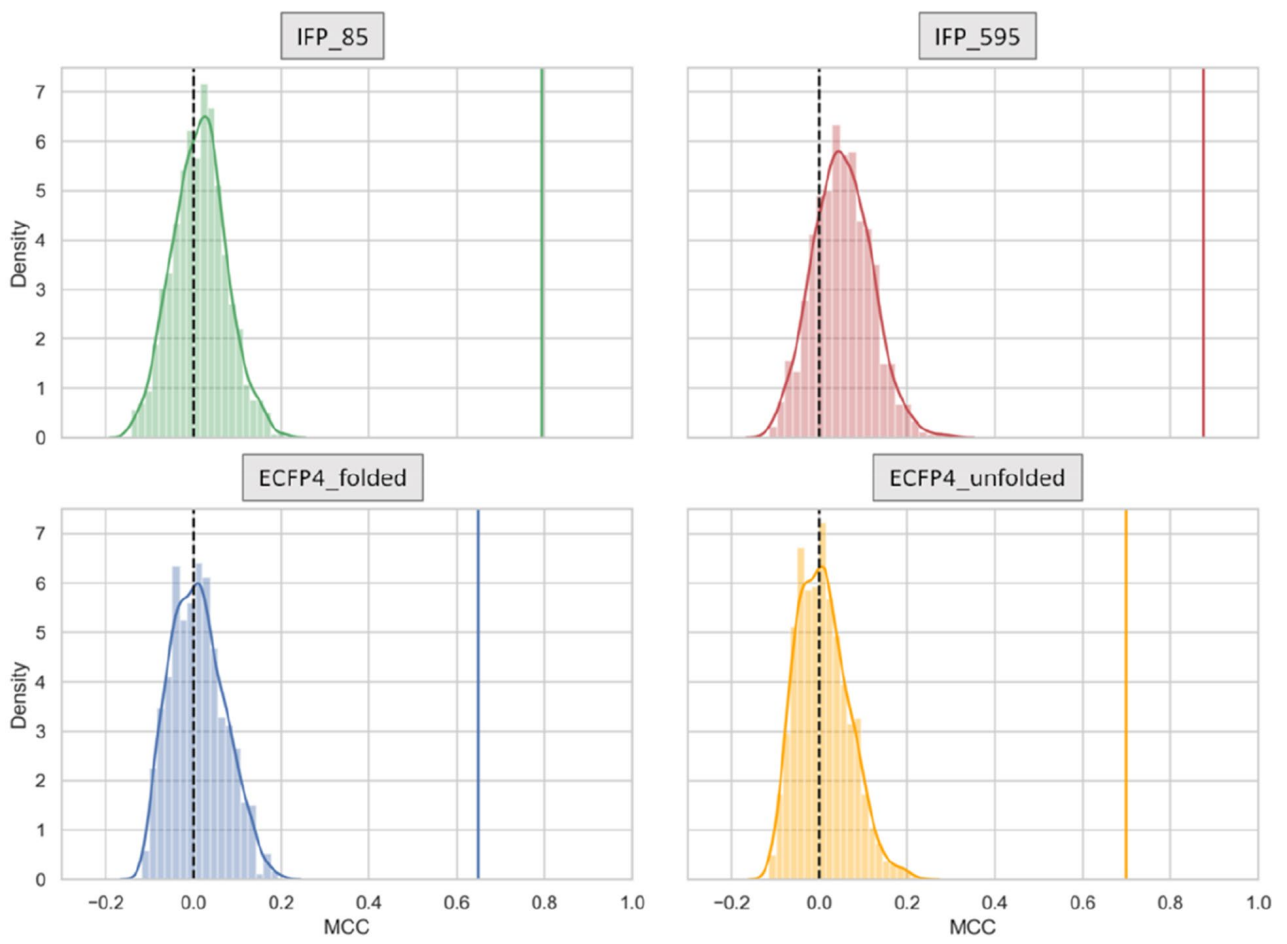
Permutation tests. For predictions on test sets, MCC value distributions are shown for RF models trained with randomized class labels using different representations. The vertical dashed line indicates MCC = 0 and the solid colored lines mark model performance for the same individual trial

As a control, permutation tests were carried out (see Methods section) to confirm that RF models indeed detected inhibitor type-specific patterns. Figure 3 shows the results of permutation tests, i.e., the distribution of MCC values for 1000 RF models trained on data with randomized (shuffled) class labels using different representations. The results show that control models had only very little predictive capacity. None of the control models approached the accuracy levels of models with non-permuted labels, which supported the significance of the results.

**Fig. 4:**
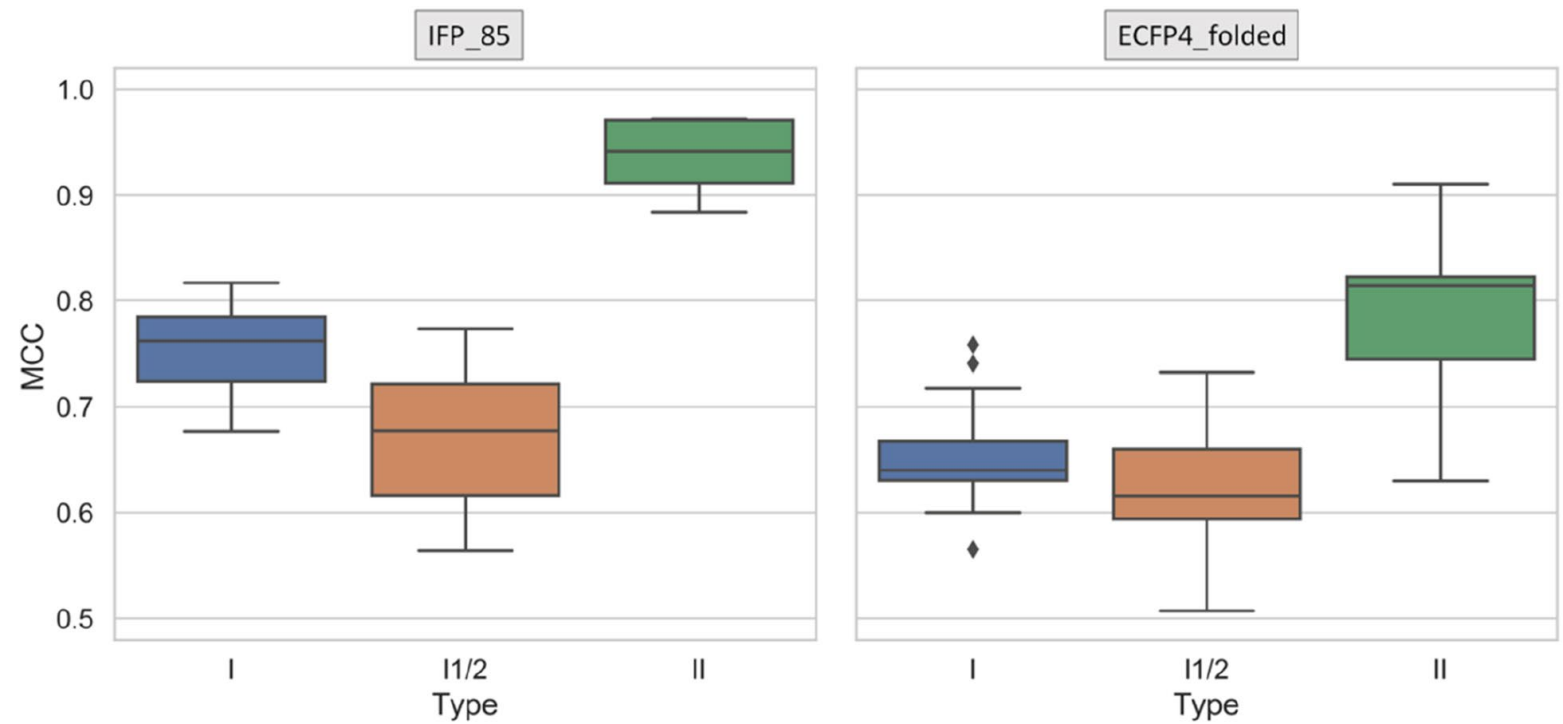
Per-class performance. MCC value distributions are separately shown for test set predictions of type I (blue), I½ (orange), and II (green) kinase inhibitors, respectively, with RF models using IFP_85 and ECFP4_folded, respectively

Figure 4 reports the per-class performance for different types of kinase inhibitor with RF models using basic fingerprint versions. Type II inhibitors were most accurately predicted especially using interaction information, with a median MCC of 0.95. Furthermore, prediction accuracy was higher for type I than type I½ inhibitors, which yielded median MCC values of 0.67 (IFP_85) and 0.63 (EFCP_folded). Thus, inhibitors with binding modes combining binding characteristics of type I and II inhibitors were most challenging to predict, as one might expect. The more accurate predictions of type II compared to type I inhibitors were likely due to the presence of unique hydrogen bonding groups present in many type II inhibitors that distinguish them from type I inhibitors [22, 23]. These signature groups or substructures and their interactions are accounted for by atom environment/fragment fingerprints and IFPs, respectively.

### Unsupervised learning for visualization

**Fig. 5:**
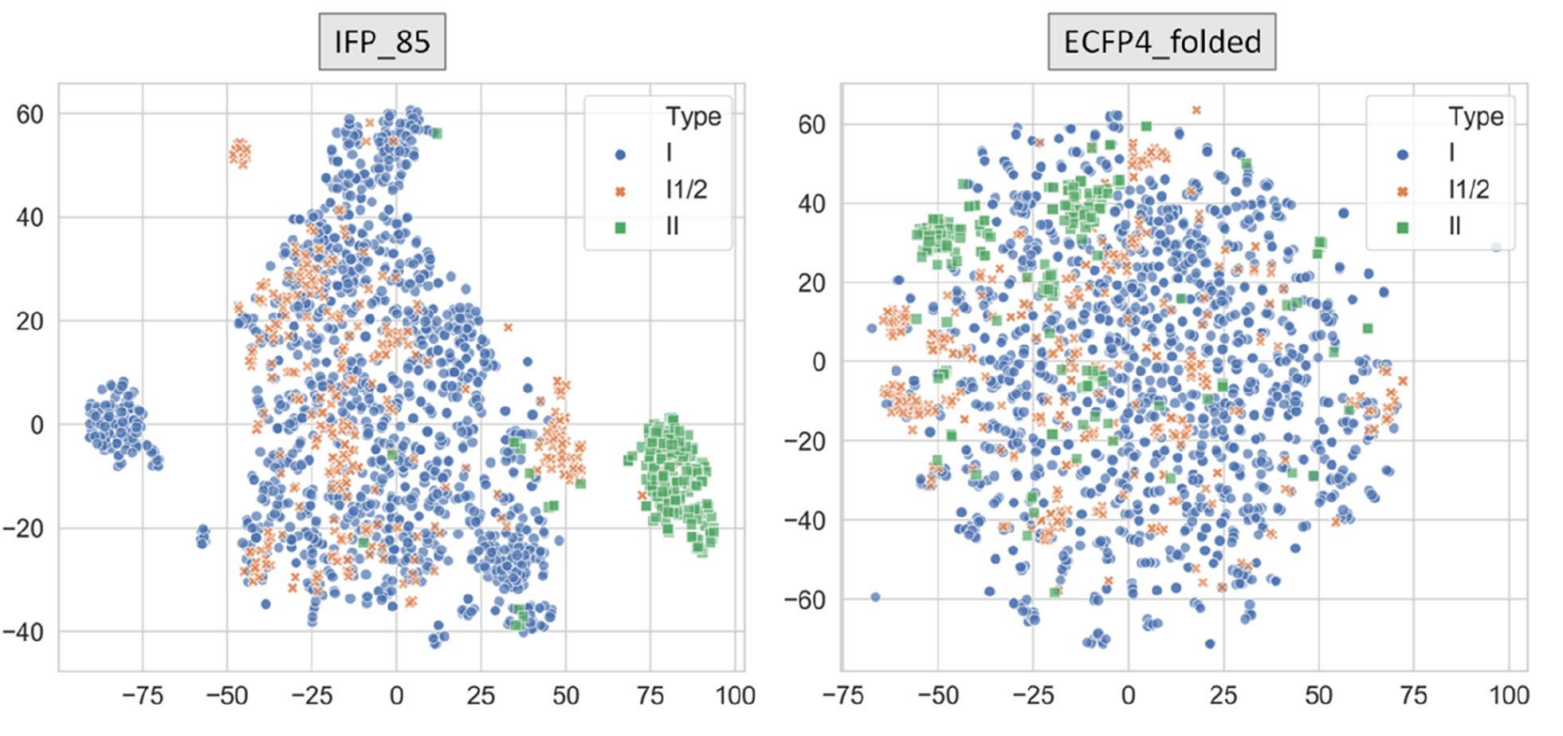
Visualization of feature spaces. Scatter plots show 2D T-SNE representations of the IFP_85 (left) and ECFP4_folded (right) fingerprint spaces on the basis of Tanimoto distances. Inhibitors (dots) are color-coded according to binding modes: type I (blue), I½ (orange), and II (green)

The unsupervised machine learning method t-distributed stochastic neighbor embedding (t-SNE) was applied for further comparison of representations and data visualization. Using this non-linear dimension reduction approach, a two-dimensional (2D) embedding was constructed from a multi-dimensional feature space on the basis of Tanimoto distances to preserve local similarities (see “Methods” section). Figure 5 shows t-SNE visualizations for IFP_85 and ECFP4_folded feature spaces containing all kinase inhibitors. The 2D t-SNE representations reveal much clearer clustering of inhibitors by type for IFP_85 than ECFP4_folded, which further prioritized IFPs for modeling. For example, t-SNE map for IFP_85 clearly separated the majority of type II inhibitors from those with other binding modes. In addition, a separate cluster of type I inhibitors of a group of phosphatidyl inositol kinases (p110a, p110d, p110g, PIK3C3, PI4KA, and PI4KB) and serine/threonine-protein kinase mTOR emerged. These kinases differ structurally from many others in the human kinome, which is also reflected by different interactions with co-crystalized inhibitors that were accounted for by IFPs. In both maps, however, type I½ inhibitors often co-localized with type I inhibitors, which also illustrated why type I½ inhibitors were overall most challenging to predict.

### Active learning

To further compare the information content of structural and interaction representations, an active learning strategy was applied combining multi-class RF modeling and entropy-based selection of training instances. RF models were iteratively built with increasing numbers of training instances for the prediction of an external test set and the remaining compound pool. While test set predictions enable the estimation of model performance, predictions of the compound pool determine the choice of instances for addition to the training set. Initially, only three compounds were randomly selected from the pool for training the first RF model (one of each inhibitor type). At subsequent iterations, 10 compounds from the pool were chosen and added for retraining the model. Compounds from the pool with the highest uncertainty in their predictions, quantified as information entropy, were selected. The information entropy concept can be applied to the predicted probabilities of three possible states: type I, I½, and II. Therefore, entropy can also be interpreted as the expected amount of information that an instance would add to the model. The model was iteratively refined and tested to optimize prediction accuracy.

**Fig. 6:**
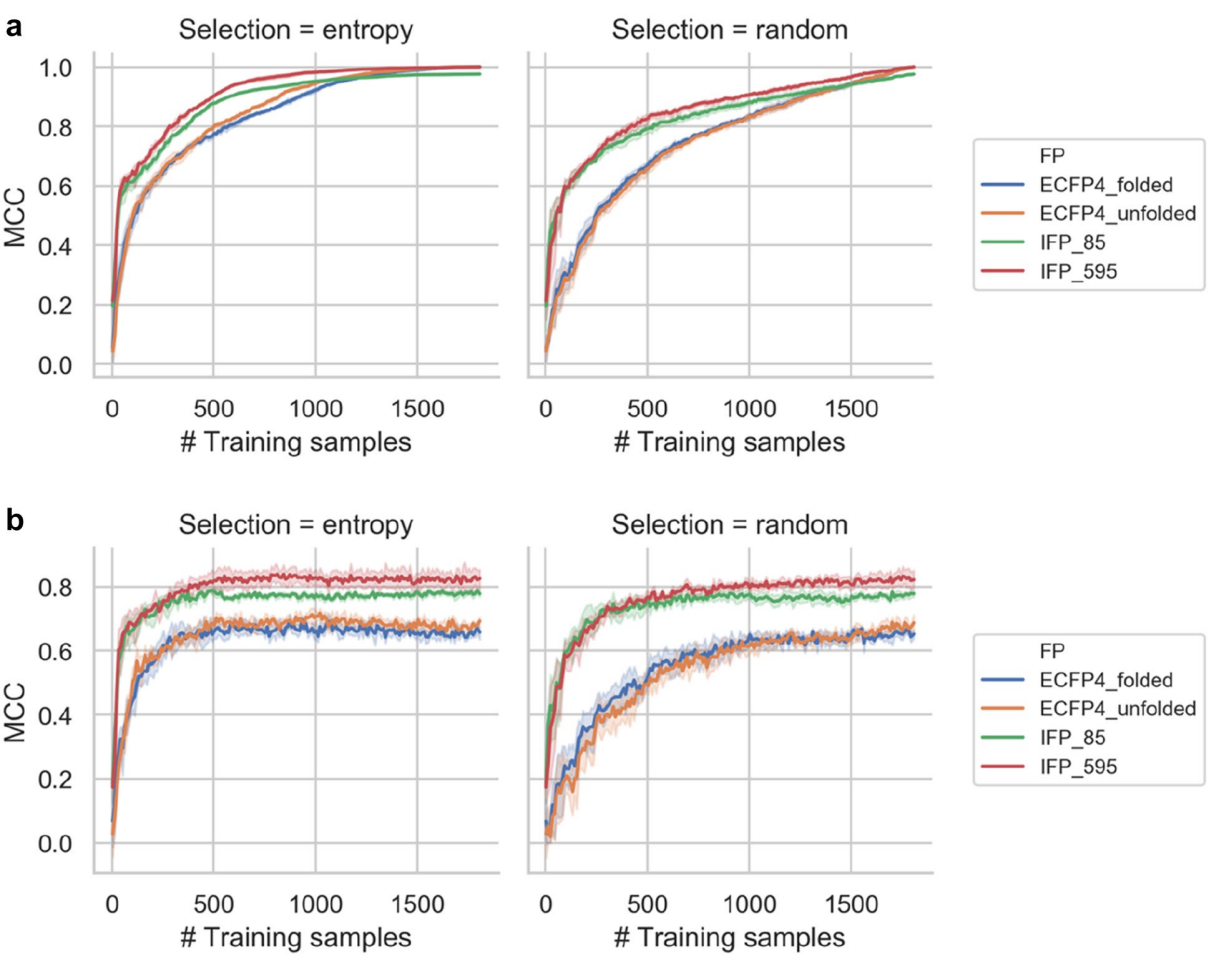
Active learning performance. The MCC values for **a** compound pool and **b** test set predictions are reported for different representations using entropy-based (left) and random (right) selection of training samples. In **b**, shaded areas of each curve indicate standard deviations of different prediction trials

Three independent trials with two-fold external cross-validation of active learning were performed. Figure 6 shows average MCC values at increasing numbers of training samples using different representations. As a control, entropy-based active learning was compared to random sample selection from the compound pool. In Fig. 6a, MCC values reported for the complete compound pool and training set. Since compound instances were iteratively added to the training set, the model predicts more instances from the training set and less from the compound pool at each interaction. At the end of this procedure, RF models were built to predict the complete training set (i.e. 90% of the total data set). These models displayed nearly perfect accuracy. The results for compound pool predictions using different representations are shown in Fig. 6a. Entropy-based selection yielded earlier optimization of MCC performance compared to random selection. Figure 6b reports MCC values for classifying the external test set. When using ~ 500 training instances, prediction performance reached a plateau with MCC values ~ 0.8 and remained constant for further increasing numbers of training samples ultimately including all pool compounds  (~ 1800). Prediction accuracy was higher for IFPs than ECFP4. For IFPs, there was a confined early improvement in MCC performance for entropy-based over random selection. By contrast, for ECFP4, the active learning entropy selection of training instances provided a significant advantage. Taken together, the results in Fig. 6 reveal that IFPs are information-rich representations with high redundancy. A high level of interaction redundancy captured by IFPs was indicated by early saturation of prediction performance using only limited numbers of training instances, even if randomly selected. Hence, small training sets already yielded sufficient IFP information for discriminating between different types of kinase inhibitors. Furthermore, high redundancy was indicated by the observation that IFP_595 only yielded a minor improvement in prediction accuracy compared to the basic IFP_85 version with no further specified interactions. Both ECFP4_unfolded and ECFP4_folded had lower information content than IFPs but higher dimensionality. For compound pool predictions with ECFP4, many more training examples than for IFPs were required for successful model building. Interestingly, for test set predictions, selection of training instances based on entropy also resulted in an early optimization of prediction performance, albeit at a lower level than IFPs. ECFP4 predictions with entropy-based selection reached a plateau at MCC values ~ 0.6.

**Fig. 7:**
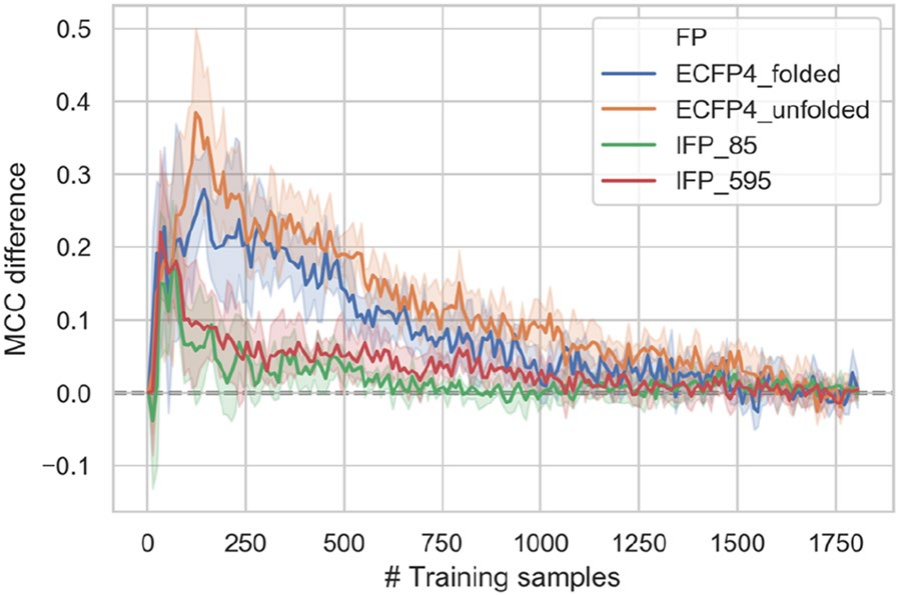
Entropy-based versus random selection. For varying training set size, the MCC value difference between entropy-based and random selection is reported for test set predictions using different representations. Shaded areas of each curve indicate standard deviations of difference calculations between corresponding predictions

Figure 7 monitors the difference between MCC values for entropy-based and random selection and increasing numbers of training instances. For each fingerprint, a performance difference peak is observed. For ECFP4_folded, the largest difference corresponded to 0.28 MCC units and occurred for ~ 140 examples. By contrast, for ECFP4_unfolded, the largest difference was 0.4 MCC units for ~ 120 training samples. For IFPs, the maximum MCC difference was ~ 0.2 for small numbers of training instances including ~ 30 (IFP_85) and ~ 60 compounds (IFP_595). These findings confirmed that selection based on entropy yielded informative training instances especially for atom environment fingerprints. For the information-rich IFPs, even random selection led to early increases in predictive performance, resulting in a small peak difference between entropy-based and random selection for small numbers of training instances.

Although IFPs capture more information about compound binding modes than atom environment fingerprints, predicting kinase inhibitor binding modes from chemical structure also produces overall accurate predictions and remains attractive for practical applications. This is the case because X-ray structures are required to generate IFPs for predicting new compound binding modes. However, once a structure with a new inhibitor is obtained, the binding mode can be directly determined, without the need to translate interactions into an IFP for machine learning. By contrast, once a compound structure-based model is trained and validated it can be readily used to predict binding modes of new inhibitors.

**Fig. 8:**
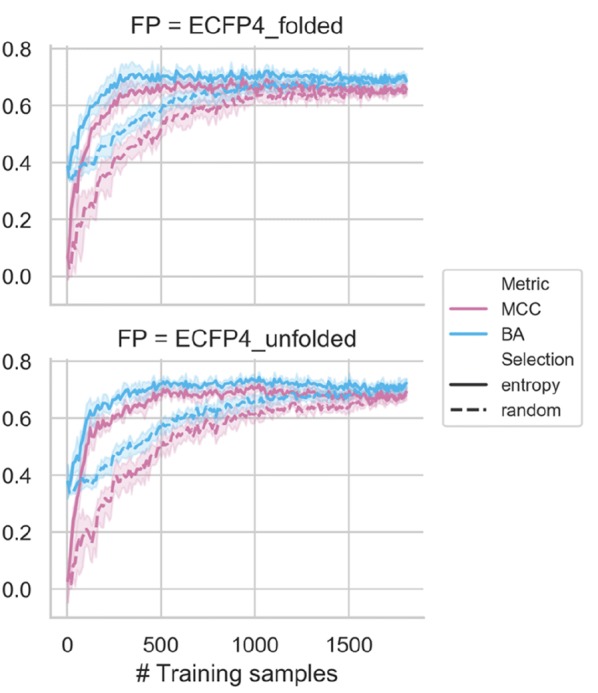
Active learning on the basis of chemical structure. Test set MCC (purple) and BA (blue) performance is shown for increasing numbers of training instances, with entropy-based (solid line) and random (dashed line) selection of compounds from the pool. Shaded areas of each curve indicate standard deviations of different prediction trials

The results in Fig. 8 indicate that on the order of 500 experimentally determined structures of inhibitor binding modes were required to maximize the accuracy of predictions using the folded as well as unfolded ECFP4 versions. For these ECFP4-based predictions, entropy-based instance selection was essential for effective active learning. The results reveal promising predictions of binding modes of test inhibitors on the basis of entropy-guided selection of training samples, with an accuracy approaching 80% for ~ 500 training compounds. Prediction performance essentially remained constant for large numbers of training instances. Hence, the number of currently available kinase inhibitors with experimentally determined binding modes by far exceeds (approx. 4-fold) the numbers of informative training instances required for overall accurate multi-class prediction of inhibitor binding modes on the basis of chemical structure.

### Feature analysis

**Fig. 9:**
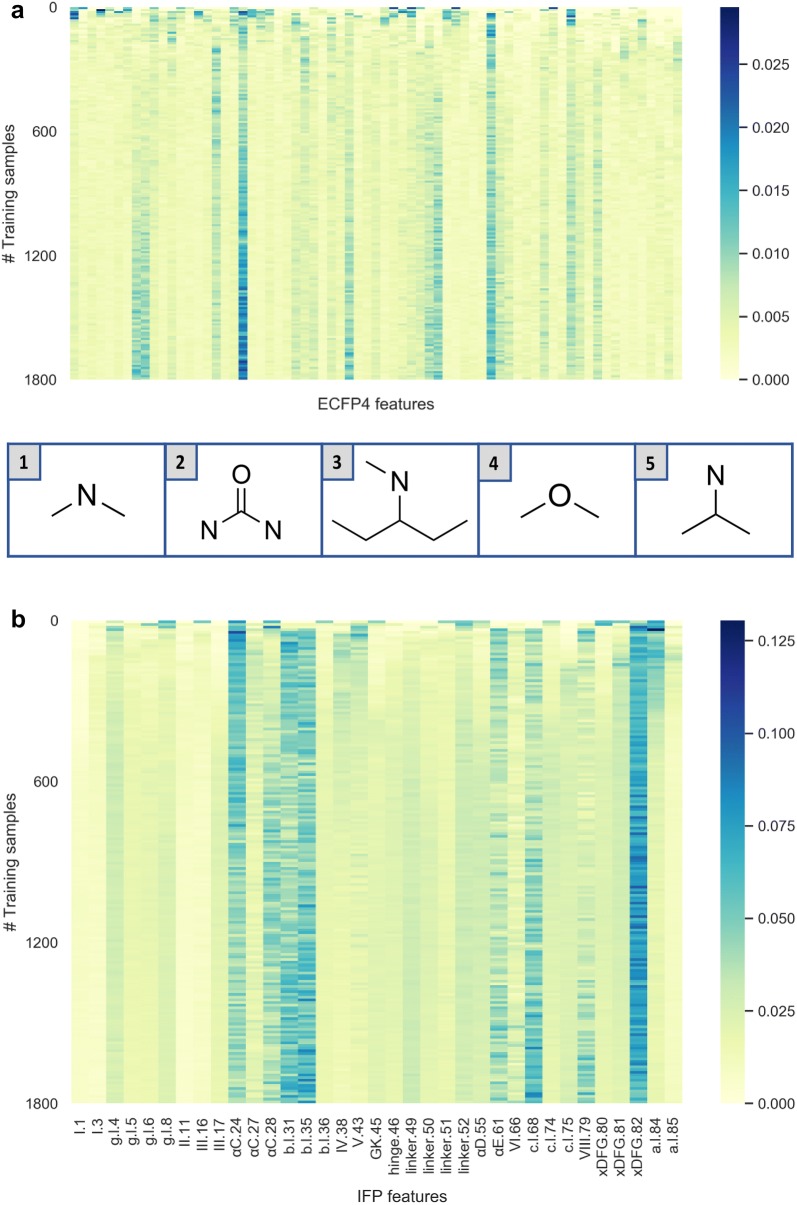
Feature importance analysis. Importance values for **a** ECFP4 and **b** 85-bit IFP features are reported for different numbers of training set samples (i.e. active learning iterations). In **a** and **b**, only features with a median importance of at least 20% and 10% of the maximum are shown, respectively. Importance values are color-coded as indicated. In **a**, the five features with largest median values across all iterations are shown in the insert at the bottom

The importance of individual IFP and ECFP4 features for the prediction of kinase inhibitor binding modes was also assessed (see Methods section). For each active learning step, a multi-class RF model was built and its feature importance values were estimated. Figure 9 shows the change in feature importance over different active learning iterations, i.e., different numbers of training set samples.

The median importance value of each feature was calculated over all iterations. In Fig. 9, features with a median importance value of at least 20% and 10% of the maximum are shown for ECFP4 and IFP, respectively. Overall, very similar feature sets were consistently prioritized when re-training the classification models. As indicated by the observed model performance, large training sets were not required to accurately predict kinase inhibitor binding modes. However, the RF algorithm detected discriminative feature patterns early on. The analysis showed that the important features detected with 90% of the data were very similar to those prioritized using smaller training sets.

Feature importance values were also assessed for RF models built with concatenated fingerprints, which included both atom environments and IFP features. In this case, features found to be most relevant for the predictions were the same IFP features as observed before. Thus, these findings revealed that the inclusion of ECFP4 features essentially retained prioritized IFP features, yielding very similar results.

## Conclusion

In this work, classical random forest models as well as active learning variants enabled the assessment of the information content of two conceptually different molecular representations for predicting compound binding modes. The predictive ability of alternative feature representations as well as their redundancy was evaluated. Ultimately, one would like to predict different binding modes on the basis of ligand structure, which is of high relevance for practical applications. However, IFP-based models were generated to put the performance of ligand-based representations into perspective and evaluate the relative information content for predictions. Successful predictions were obtained with both ECFP4 and IFPs. Moreover, the performance on the basis of both representations was significantly better than expected by chance as assessed with a random classifier. IFPs showed consistently superior predictive performance than chemical fingerprints, which reflected larger information content of IFPs, especially for the high-resolution version IFP_595. Nonetheless, ECFP4 yielded successful predictions that generally differed by less than 0.2 MCC units. An active learning strategy based on multi-class RF and entropy-based instance selection was introduced and the results indicated the suitability of this approach for limiting the data required to accurately predict binding modes of kinase inhibitors. Entropy-based selection of training compounds strongly influenced predictions on the basis of ECFP4, having lower intrinsic information content. Active learning revealed that ~ 25% of the available training samples were sufficient to reach near maximal MCC values. For practical applications, predicting binding modes of newly discovered kinase inhibitors from chemical structure is particularly attractive.

## Methods

### Data set

Kinase inhibitors with different binding modes were extracted from the KLIFS database [6, 7] as described [18], which organizes these inhibitors on the basis of structural information from kinase-inhibitor complexes. Binding modes were assigned on the basis of conformational states observed for the DFG motif and αC-helix in each kinase-ligand complex structure. Conformational state information was obtained from KLIFS using the open source virtual machine 3D-e-Chem-VM. Inhibitors with different binding modes were assembled, except allosteric and covalent inhibitors, which were only available in small numbers and for which IFPs could not be computed in a consistent manner. In addition, small numbers of kinase inhibitors capable of adopting multiple binding modes were not selected. A total of 2008 kinase inhibitors were obtained including 1424 type I, 394 type I½, and 190 type II inhibitors (Table 1), which originated from 2288 X-ray structures (representing a subset of inhibitors previously reported inhibitors [18]).

### Feature representations

#### Interaction fingerprints

**Fig. 10:**
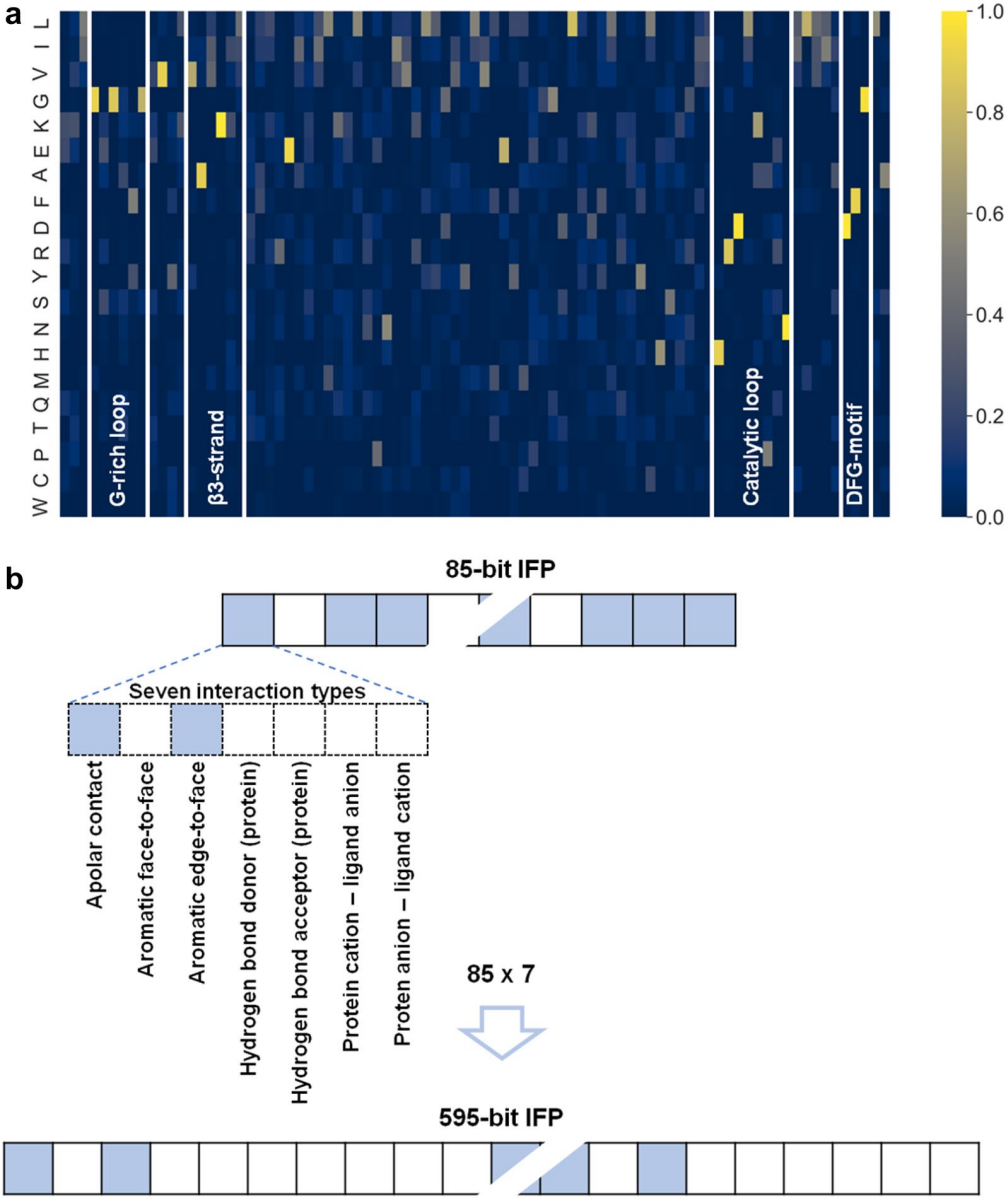
Kinase binding site representation and IFPs. **a** For 85 residues positions comprising the kinase binding site region (horizontal axis), the amino acid ratios across all kinase structures (vertical axis) is reported as a frequency-based color gradient heatmap. Key structural elements are indicated. **b** The expansion of the 85-bit vector to the 595-bit IFP through specification of seven different types of interactions is illustrated

The KLIFS database defines a set of 85 sequence-discontinuous residue positions forming the kinase binding site region where kinase-ligand interactions with type I½, II, and III inhibitors take place. A bit vector recording the presence or absence (“on” or “off”) of ligand interactions with each of these 85 positions (where residues might differ) was used as a basic IFP representation (IFP_85). The frequency or occurrence of amino acid residues at each position across all 2288 X-ray structures used in the analysis is provided in Fig. 10a. The basic 85-bit vector was further extended by generating a 595-feature IFP by assigning interactions involving each residue to seven different categories according to Fig. 10b, permitting multiple interactions per residue (IFP_595). For inhibitors with IFPs for different X-ray structures, a consensus IFP was calculated by determining the majority of “on” or “off” records. In case of a tie, the interaction was set “on”. Following these procedures, for each inhibitor, a final (unique or consensus) 85-bit and 595-bit IFP were generated using KLIFS.

#### Atom environment/fragment fingerprints

For each inhibitor, ECFP4 [23] was calculated using an in-house Python script based on OEChem [24]. ECFP4 enumerates layered atom environments up to the given diameter and encodes them as integers using a hashing function [23]. These atom environments constitute a feature set of variable size that can be folded to a fixed length (1024 bits) through modulo mapping. Both ECFP4_folded and ECFP4_unfolded were investigated.

### Random forest algorithm

RF is a machine learning algorithm that consists of an ensemble of decision trees [25]. Each tree applies recursive partitioning and represents a sequence of binary decisions on the basis of feature values. To avoid the generation of correlated trees, individual trees were built using bootstrap aggregating and feature bagging [26]. For a given test compound, feature values indicate the decision path in a tree until reaching a leaf node. Each leaf node is characterized by a number of training instances sharing the same feature decision path. The majority class is selected as prediction outcome for a test instance. Next, final predictions are determined by the consensus decision across trees in the ensemble. A multi-class RF was implemented to distinguish between type I, I½, and II inhibitors. For this prediction task, RF assigns each compound to a single class or binding mode. The predicted class corresponds to the binding mode with largest proportion of training instances at a given leaf node. For RF generation, scikit-learn was used [27]. The number of trees was set to 100, class weights were applied to account for class imbalance, and default settings were considered for other hyper-parameters. Feature importance was calculated for RF models.

The estimated importance of a feature for a node split is the improvement in the split criterion, which needs to be separately accumulated for each feature over all decision trees comprising the RF [28]. The implemented RF classifier was based on the Gini impurity criterion. Thus, feature importance values were calculated as the mean decrease in node impurity weighted by the probability of reaching a given node [28].

### Active learning strategy

Active learning combines a machine learning model such as RF with the iterative selection of informative training instances to retrain and further improve the predictive model [29]. In the context of binding mode prediction, active learning classifies kinase inhibitors according to their type and decides which training instance(s) to select next. In this study, training instances were selected from a compound pool that representing different experimental outcomes. To select informative training instances, it was simulated that binding modes inhibitors from the pool were unknown until they were predicted and incorporated to the evolving training set. The active learning strategy applied here consisted of a multi-class RF model and entropy-based data selection.

Shannon entropy is a concept from information theory [30]. Information entropy quantifies uncertainty and is defined by the following expression.

$$H = - \mathop \sum \limits_{i} p_{i} \log_{2} p_{i}$$

where $p_{i}$ is the probability of the state *i* (or a given binding mode). Here, possible states include type I, type I½ and type II inhibitors. Accordingly, instance selection is based on the uncertainty of the current RF model to predict the binding mode of kinase inhibitors in the pool. Therefore, *H* is calculated for individual predictions of the ensemble classifier.

### Calculation protocols

The calculation set-up for active learning is illustrated in Fig. 1 and begins with stratified data splitting into a compound pool (90%) and test set (10%). The split was carried out per activity class to ensure the presence of same class distribution in the training and test sets. In the first iteration, three instances (one per class) are randomly selected and used to train the initial RF model. In subsequent iterations, a number of compounds (N) from the compound pool are selected based on information entropy from RF predictions. N cases with largest entropy across their predictions, reflecting high model uncertainty, are added to the training set. Small N values increase computational costs due to more required cycles of model retraining while large N values may lead to information redundancy. As a desirable trade-off between model retraining and batch size, N was set to 10 for all active learning trials. Results were averaged across six independent trials, resulting from two independent compound pool/test set splits with three executions each with random selection of the first three instances. Standard RF models were also built with distinct feature representations. In this case, 20 independent trials were performed with 90% of the data for training and 10% for testing.

The 90%/10% data splits were applied to generate a large compound pool for active learning. The potential influence of overfitting of individual models was minimized by estimating performance on the basis of cross-validation. As a control, the calculations were repeated on the basis of 70%/30% data splits and the results were found to closely correspond to those reported above.

### Performance assessment

Model performance was assessed using MCC [31] and BA [32], as defined below:

$${\text{MCC}} = \frac{{{\text{TP}} \times {\text{TN}} - {\text{FP}} \times {\text{FN}}}}{{\sqrt {\left( {{\text{TP}} + {\text{FP}}} \right)\left( {{\text{TP}} + {\text{FN}}} \right)\left( {{\text{TN}} + {\text{FP}}} \right)\left( {{\text{TN}} + {\text{FN}}} \right)} }}$$

$${\text{BA}} = \frac{1}{2}\left( {\frac{\text{TP}}{{{\text{TP}} + {\text{FN}}}} + \frac{\text{TN}}{{{\text{TN}} + {\text{FP}}}}} \right)$$

where TP, TN, FP, FN refer to true positives, true negatives, false positives and false negatives, respectively.

In addition, permutation-based *p*-values were calculated to assess performance significance [33]. Permutation tests were performed for one individual trial, i.e. a single 90% and 10% data split. Therefore, 1000 RF models were trained on the 90% of the data with randomly shuffled labels and the performance was estimated on the test set (10%). *p*-values account for the number of models with shuffled labels that yield at least the same performance as the RF derived from training instances with original labels. Thus, in this case, the smallest achievable *p*-value is 1/1000.

### T-distributed stochastic neighbor embedding

For data exploration and visualization, t-SNE was used [27, 34]. T-SNE is a non-linear dimension reduction method that generates low-dimensional representations preserving the local similarity between data points in the original space. Pairwise distances between compounds are calculated first and then converted to conditional probabilities. Therefore, a normal distribution centered at each point is assumed and the density of points is determined to account for probability-based local similarity. Accordingly, conditional probabilities are large for instances that are close to each other and small for distant instances. The resulting structure is replicated in lower-dimensional space by minimizing the Kullback–Leibler divergence [35] between joint probabilities in higher- and lower-dimensional space. In this study, Tanimoto distance [36] was used as a distance measure and a 2D embedded space as the low-dimensional representation. Different perplexity values were examined revealing very little influence on the visualizations, and perplexity was constantly set to 30.

## Data Availability

The kinase inhibitor data including kinase annotations for all compounds are publicly available in an open access deposition [37]. In addition, compound data sets and scripts including a Jupyter notebook with the active learning method are freely available for download via the following link: https://uni-bonn.sciebo.de/s/EH2ieO4T107WXxf.

## Abbreviations

BA: Balanced accuracy
ECFP: Extended connectivity fingerprint
FN: False negatives
FP: False positives
H: Information entropy
IFP: Interaction fingerprint
MCC: Matthew’s correlation coefficient
RF: Random forest
TN: True negatives
TP: True positives
